# Differential vascular endothelial cell toxicity of established and novel BCR-ABL tyrosine kinase inhibitors

**DOI:** 10.1371/journal.pone.0294438

**Published:** 2023-11-20

**Authors:** Yihua Wang, Richard J. Travers, Alanna Farrell, Qing Lu, Jennifer L. Bays, Alec Stepanian, Christopher Chen, Iris Z. Jaffe

**Affiliations:** 1 Molecular Cardiology Research Institute, Tufts Medical Center, Boston, MA, United States of America; 2 Tufts University, Medford, MA, United States of America; 3 The Biological Design Center and Department of Biomedical Engineering, Boston University, Boston, MA, United States of America; 4 The Wyss Institute for Biologically Inspired Engineering, Harvard University, Boston, MA, United States of America; Osmania University, Hyderabad, India, INDIA

## Abstract

BCR-ABL tyrosine kinase inhibitors (TKIs) have dramatically improved survival in Philadelphia chromosome-positive leukemias. Newer BCR-ABL TKIs provide superior cancer outcomes but with increased risk of acute arterial thrombosis, which further increases in patients with cardiovascular comorbidities and mitigates survival benefits compared to imatinib. Recent studies implicate endothelial cell (EC) damage in this toxicity by unknown mechanisms with few side-by-side comparisons of multiple TKIs and with no available data on endothelial impact of recently approved TKIs or novels TKIs being tested in clinical trials. To characterize BCR-ABL TKI induced EC dysfunction we exposed primary human umbilical vein ECs in 2D and 3D culture to clinically relevant concentrations of seven BCR-ABL TKIs and quantified their impact on EC scratch-wound healing, viability, inflammation, and permeability mechanisms. Dasatinib, ponatinib, and nilotinib, the TKIs associated with thrombosis in patients, all significantly impaired EC wound healing, survival, and proliferation compared to imatinib, but only dasatinib and ponatinib impaired cell migration and only nilotinib enhanced EC necrosis. Dasatinib and ponatinib increased leukocyte adhesion to ECs with upregulation of adhesion molecule expression in ECs (ICAM1, VCAM1, and P-selectin) and leukocytes (PSGL1). Dasatinib increased permeability and impaired cell junctional integrity in human engineered microvessels, consistent with its unique association with pleural effusions. Of the new agents, bafetinib decreased EC viability and increased microvessel permeability while asciminib and radotinib did not impact any EC function tested. In summary, the vasculotoxic TKIs (dasatinib, ponatinib, nilotinib) cause EC toxicity but with mechanistic differences, supporting the potential need for drug-specific vasculoprotective strategies. Asciminib and radotinib do not induce EC toxicity at clinically relevant concentrations suggesting a better safety profile.

## Introduction

Chronic myeloid leukemia (CML) is caused by the Philadelphia chromosome (9:22) translocation in myeloid leukocytes. This translocation produces the constitutively active BCR-ABL tyrosine kinase which drives proliferation of malignant cells [1]. The development of BCR-ABL-targeted tyrosine kinase inhibitors (TKIs) has dramatically improved survival from approximately 30% to greater than 70% at 5 years [2]. BCR-ABL TKIs have thus transformed CML from a highly lethal disease treated with chemotherapy and allogeneic stem cell transplantation into a chronic illness with excellent long-term survival. As a result, CML prevalence is increasing and expected to approach 200,000 people in the US by 2050 [3,4].

Despite this clinical success, only 50–60% of patients achieve the treatment milestone of major molecular response at one year when treated with the first generation BCR-ABL TKI imatinib, due to either incomplete response or development of resistance [5]. As such, newer generation BCR-ABL TKIs continue to be developed, including dasatinib, bosutinib, nilotinib, and ponatinib, which achieve one year major molecular response rates of 75–100%, even in patients resistant to imatinib [5,6]. Thus, the current standard of care for intermediate or high risk CML is initiation of one of these second or third generation TKIs at the time of diagnosis [7]. Furthermore, approximately 5% of patients who develop resistance to imatinib will harbor a T315I mutation in the BCR-ABL gene, which confers resistance to almost all BCR-ABL TKIs, spurring continued development of new agents. CML with T315I was only responsive to ponatinib until the recently approval of asciminib in 2021 [8–10]. Multiple additional BCR-ABL TKIs are currently in development including bafetinib which has shown activity in imatinib-resistant CML, high grade gliomas and prostate cancer [11,12], and radotinib which was approved in Korea in 2015 as first line therapy for CML [13].

Despite the success of newer agents, follow up to cancer trials has revealed that the benefit in cancer survival with the second and third generation BCR-ABL TKIs compared to imatinib is limited by cardiovascular toxicities [6]. Specifically, dasatinib, nilotinib, or ponatinib are associated with a 3- to 4-fold increased risk of acute arterial thrombotic events including myocardial infarction (MI), stroke or acute limb ischemia compared to imatinib [6,14]. For example, in the PACE trial, 25% of ponatinib-treated patients developed acute arterial thrombotic events, which included myocardial infarction (MI), stroke, or critical limb ischemia. Furthermore, in the PACE trial the relative risk of adverse cardiovascular events was 2.2 fold higher for patients with at least one cardiovascular risk factor compared to those with no risk factors, suggesting synergy between pre-existing cardiovascular risk and TKI treatment [15]. Since the average age of presentation for CML is 64 years old, 64% of CML patients have a history of hypertension, diabetes, or known atherosclerotic heart disease at the time of initiation of treatment with a second or third generation BCR-ABL TKI [16].

The pathophysiologic mechanisms behind the acute arterial occlusive events that occur with second and third generation BCR-ABL TKIs are unknown. While there is some conflicting data as to whether ponatinib and nilotinib might increase platelet reactivity (reviewed in 15, [17–19]), emerging evidence suggests that vascular endothelial cell (EC) damage and dysfunction likely contributes [19–23]. The healthy endothelium is anti-inflammatory and anti-thrombotic, preventing pathologic leukocyte and platelet adhesion and thrombosis formation. When the endothelium is damaged, by traditional risk factors, mechanical injury or by drug toxicity, the damaged endothelium exposes the pro-thrombotic vessel basement membrane to circulating platelets which can initiate acute arterial thrombosis. As such, factors that induce EC death, denudation or impair healing mechanisms may contribute to the increased risk of arterial thrombosis [24]. EC injury also results in pro-inflammatory changes including expression of cellular adhesion molecules like intercellular and vascular adhesion molecules (ICAM1, VCAM1) or E- and P-selectin which induce leukocyte and platelet adhesion to the vasculature [25,26]. Furthermore, vascular inflammation is an early step in the development of atherosclerotic plaques and importantly, the degree of inflammation associates with the risk of plaque rupture, the most common cause of life threatening arterial thrombosis including stroke and MI [27]. Thus, endothelial damage may contribute to acute arterial thrombosis and would be expected to synergize with traditional cardiovascular risk factors, which also induce EC damage.

All the BCR-ABL TKIs inhibit ABL kinase, but they also have divergent off target effects. Whether the different BCR-ABL TKIs impact EC function in the same or different ways is not clear. Given the long duration between start of treatment and development of cardiovascular events, it will be years before cardiovascular safety data is available for new drugs like asciminib, radotinib, and bafetinib. Thus, *in vitro* assays that can determine the potential vascular impact and predict side effects of these agents prior to long term clinical data could improve drug development and safety. Here we provide a side-by-side comparison of imatinib, dasatinib, ponatinib, nilotinib, and three new agents asciminib, radotinib and bafetinib at concentrations up to the maximum plasma concentration (Cmax) to which the endothelium is exposed in human clinical trials. We compare the impact of each drug on EC wound healing, inflammation and permeability. To elucidate mechanisms, we also investigate the relative effect of each drug in assays for cell migration, proliferation, viability, necrosis, adhesion molecule expression, and cell junction integrity of human engineered microvessels (hEMVs) [28]. We identify similarities and differences in the toxicity mechanisms of dasatinib, ponatinib, and nilotinib in human ECs and show for the first time that asciminib and radotinib do not damage ECs in these assays while bafetinib demonstrates some degree of EC toxicity.

## Methods

### Cell culture and drug concentrations

Human umbilical vein endothelial cells (HUVECs) (Lonza catalogue number C2519A) and human monocytic U937 cells (ATCC CRL-1593.2, pro-monocytic, human cell line derived from the histiocytic lymphoma of a 37 year old male source) were cultured as previously described [29,30]. HUVECs were chosen based on prior studies demonstrating that toxicity of BCR-ABL TKIs in HUVECs correlates with and predicts arterial thrombotic events in humans [23]. For all experiments, HUVECs were treated with vehicle (dimethyl sulfoxide, DMSO) or each TKI at the steady-state peak plasma concentrations reported in CML patients: imatinib (4.0 μM) [31], dasatinib (0.2 μM) [32], ponatinib (0.1 nM) [8], nilotinib (3 μM) [33], asciminib (1.1 μM) [9], radotinib (0.38 μM) [34], and bafetinib (1.0 μM) [12]. Viability assays were also performed at 0.5 Cmax and 0.25 Cmax to assess the dose-response relationship of EC toxicity. BCR-ABL TKIs were maintained at 1000x concentration in DMSO at -80°C and diluted into growth media immediately prior to use. DMSO was used at 1:1000x dilution as well, ensuring that the same concentration of DMSO was present in all experimental groups.

### Crk-like proto-oncogene (CRKL) immunoblotting

CRKL, a known ABL-kinase phosphorylation target, was used to confirm ABL-kinase inhibition by each drug in ECs. HUVECs were cultured to 80% confluences in a 12 well plate, then treated for 2 hours with vehicle or BCR-ABL TKI at the Cmax concentrations indicated above at 37°C. Cells were then washed 2x with warm PBS and harvested with direct addition of 150 μL of 1x denaturing sample buffer (Boster). Samples were boiled at 95°C for 5 minutes, separated via SDS-PAGE on 4–15% gradient Mini-Protean TGX gels (BioRad), transferred to Immobilion-FL PVDF membranes (Millipore), blocked for 1 hour in Intercept Blocking Buffer (Licor), and then incubated with rabbit anti CRKL or rabbit pY207 phospho-CRKL (both 1:500 dilution, Cell Signaling Technologies) in Intercept Blocking Buffer (LiCor) + 0.2% Tween20 overnight at 4°C. Blots were then washed 4x with TBS+0.2% Tween 20 (TBST) and incubated with donkey anti rabbit conjugated to DyLight 800 secondary antibody (1:10,000, LiCor) in Intercept Blocking Buffer + 0.2% Tween 20 + 0.02% SDS for 1 hour at room temperature, washed 4x with TBST and imaged (Odyssey M imager, LiCor). Mouse anti B-Actin (1:1000, Sigma) detected with donkey anti-mouse conjugated to DyLight 800 (Licor) was used as loading control. Protein bands were quantified with Empiria software from LiCor and the ratio for phosphorylated to total CRKL was calculated.

### Scratch wound healing assay

HUVECs were cultured to approximately 90% confluence and then scratched with a sterile pipette tip to create a wounded area lacking ECs. Scratched HUVEC monolayers were incubated in medium containing vehicle or TKI in 4% bovine growth serum (BGS, Cytiva Life Sciences) with 2 μM 5-ethynyl-2’-deoxyuridine (EdU, Thermo Fisher). After 18 hours cells were fixed in 4% paraformaldehyde for 30 minutes at room temperature, permeabilized with 0.1% Triton in PBS for 30 minutes at room temperature, and stained with Click-iT Alexa 488 and Hoechst DNA counterstain according to manufacturer’s instructions (Click-iT EdU Alexa 488 Imaging Kit, Thermo Fisher). Cells were imaged at 100x with a Nikon Ti Eclipse Microscope with Coolsnap EZ fluorescent camera and the total number of cells, the number of EdU+ cells (proliferated cells) and the number of EdU- cells (migrated cells) in the wound were counted by a blinded investigator in 4 fields per well and averaged for each well. 5–10 independent experiments were performed per condition.

### Cell viability assays

HUVECs were grown in a 12 well dish to 90% confluence and then treated with either DMSO or each BCR-ABL TKI for 24 hours. At 24 hours, Calcein-AM (marks viable cells with intact intracellular esterase activity), Propidium Iodide (marks dead cells lacking membrane integrity), and Hoechst (marks all cells) were diluted in a 1:1 mixture of PBS and growth media according to manufacturer’s instructions (Live Dead Cell Viability Assay Kit, Sigma) and added to the wells. The wells were then imaged at 100x on a Nikon Ti Eclipse Microscope with Coolsnap EZ fluorescent camera with appropriate filter settings. The total number of cells per field was counted via automated object counting for Hoechst stained nuclei in NIS-Elements Software (Nikon). The number of calcein-AM positive (viable) and propidium iodide positive (necrotic) cells were also determined with automated counting. The percent calcein-AM and propidium-iodide positive cells for six fields were averaged for each well. The experiment was performed with a total of 6 independent replications.

### Leukocyte adhesion assay

HUVEC, U937, or both cell lines were treated with DMSO control or each TKI for 20 hours. U937 leukocytes were labeled with BCECF-AM (Thermo Fisher) and incubated with HUVECs at room temperature for two hours and then washed off as previously described [29]. Adherent leukocytes were counted by a blinded investigator in 5 fields per well at 100x magnification with a Nikon Ti Eclipse Microscope with Coolsnap EZ fluorescent camera. 3–4 independent experiments were performed for each drug under each of the three conditions (TKI-treated HUVECs; TKI-treated U937 cells; or both cell types TKI-treated).

### Adhesion molecule immunoblotting

HUVECs or U937 cells were treated with vehicle or each TKI for 24 hours in a 12 well plate. Cells were then washed twice with PBS and harvested in 100 μL of 1x SDS-PAGE sample buffer. Cell lysate was run on an SDS-PAGE gel, transferred to a PVDF membrane (Millipore) and blocked in 5% milk in PBS. Primary antibodies used for immunoblotting were rabbit polyclonal anti-ICAM-1 (1:500, Boster Bio), rabbit monoclonal Anti-CD62P (P-selectin, 1:500, Boster Bio), VCAM1, E-Selectin, PSGL1 (1:500, Santa Cruz Biotechnology), and GAPDH (1:1000, Cell Signaling Technology) diluted in 0.5% Milk in PBS. They were then incubated with anti-rabbit IgG HRP-linked secondary antibody (1:1000, Cell Signaling Technology) diluted in 0.5% Milk in PBS and developed with ECL Western Blotting Substrate (Thermo Fisher) and quantified with ImageJ software.

### Human engineered microvessel (hEMV) fabrication

Fabrication of the human engineered microvessel (hEMV) platform has been previously described in detail [28]. Briefly, polydimethylsiloxane (PDMS) microfluidics devices were generated based on a silicone master, media reservoirs and collagen gel sites were cut out, and the microvessel channel was formed using a steel acupuncture needle inserted prior to adding bovine collagen (PureCol, Advanced BioMatrix) to cure in the mold. Cultured HUVECs (Lonza) were collected using 0.05% Trypsin-EDTA, centrifuged at 1000g for 4 min and re-suspended at 0.5 × 10^6^ cells/mL in EGM2. The cell suspension was perfused into the hEMV device and cells were allowed to adhere to the patterned channel for 10 min before washing channels with EGM2. Devices were placed in an incubated rocker and assayed 48 hours after seeding.

### hEMV permeability analysis

hEMVs were perfused with EGM2 containing TKIs or DMSO at the Cmax concentration for 3 hours prior to measuring permeability as described previously [28]. Permeability was measured by perfusing 70 kDa Texas Red conjugated dextran (Thermo Fisher Scientific) into hEMVs. Devices were imaged on a Yokogawa CSU-21-Zeiss Axiovert 200 M inverted spinning-disk microscope using a Zeiss x10 air objective and an Evolve EMCCD camera (Photometrics). Images were captured every 5 seconds for 2 minutes. Permeability coefficients of hEMVs were calculated using ImageJ and MATLAB image analysis as detailed in [28].

### hEMV Immunocytochemistry and Junctional / Cytoskeletal rearrangement quantification

Following treatment with BCR-ABL TKIs as above, hEMVs were permeabilized and fixed with 1% paraformaldehyde, 0.03% Triton X-100 in PBS at 37°C for 90 seconds and then fixed in 4% paraformaldehyde in PBS at 37°C for 20 minutes. They were then rinsed with PBS, permeabilized with 0.1% Triton X-100 in PBS for 5 mins, blocked with 2% BSA in PBS overnight at 4°C on a rocker, and stained with primary and secondary antibodies in 2% BSA in PBS overnight at 4°C with a four-hour room temperature PBS wash between primary and secondary antibody staining. Antibodies used are; VE-Cadherin antibody (F-8, sc-9989, 1:1000, Santa Cruz Biotechnology), Alexa Fluor 568 goat anti-mouse secondary (1:300, ThermoScientific). F-actin was probed with Alexa Fluor 488-labelled phalloidin (1:500, Life Technologies). Cell nuclei were stained with Hoechst 33342 Solution (20 mM) (1:1000, ThermoScientific). Samples were imaged on a Yokogawa CSU-21-Zeiss Axiovert 200 M inverted spinning-disk microscope using a Zeiss x20 air objective and an Evolve EMCCD camera (Photometrics).

To quantify junctional area of hEMVs, images were acquired through the bottom half to the midplane of each hEMV from samples stained for VE-Cadherin. In ImageJ, the 3-D image stack was reduced to 2-D with a sum slices projection and background was subtracted (rolling ball radius 100). A manual threshold was then used to generate regions of interest (ROI) defining area of junctional VE-cadherin. For each hEMV, three 100 by 100-micron ROIs along the vessel were randomly generated for area quantification (i.e. number of pixels). Area of junctional VE-cadherin was quantified for 6 hEMVs samples per condition across at least three independent experiments.

Using the same images and ROI used to calculate junctions, the levels of junctional cytoplasmic actin was quantified by measuring the fluorescence intensity of Alexa Fluor 488 phalloidin. Fluorescence intensity of junctional actin was defined as the integrated fluorescence signal within the ROI. Fluorescence intensity of non-cortical actin was defined as the total integrated fluorescence signal of the image minus the cortical actin integrated fluorescence intensity. Cortical to non-cortical actin intensity ratios were quantified for 6 hEMVs samples per condition across at least three independent experiments.

### Statistics

Statistical analysis was performed in GraphPad Prism version 9. Data for all assays were analyzed by one-way ANOVA with Dunnett’s post-hoc test if all variances were equal. If unequal variances, Brown-Forsythe ANOVA testing was used with Dunnett’s T3 multiple comparison testing. Forall assays, multiple-comparison post-test compared the degree of ABL-kinase inhibition of each drug to DMSO vehicle control.

## Results

### BCR-ABL TKIs reduce ABL kinase activity in HUVECs

**Fig 1:**
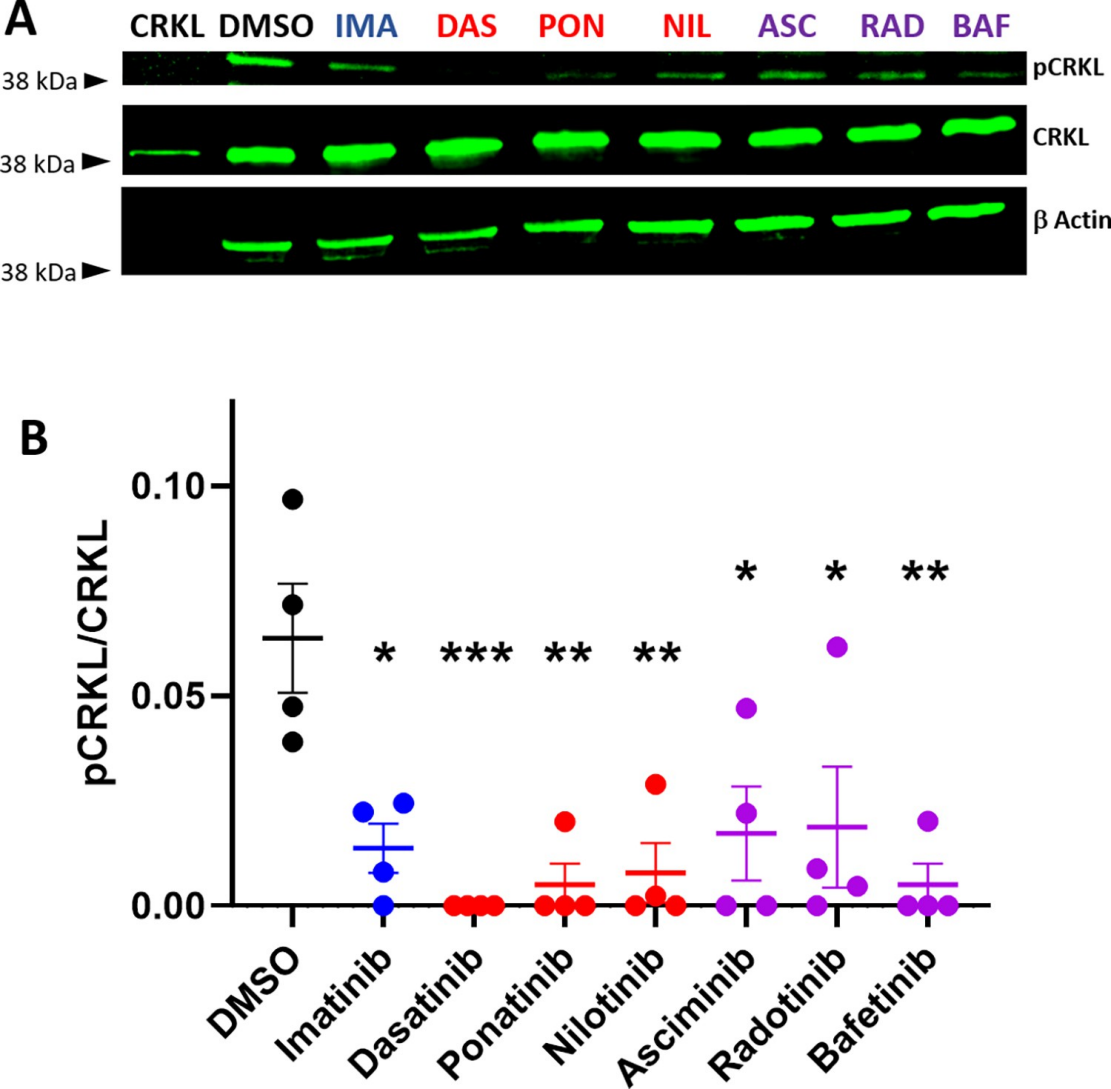
BCR-ABL TKIs inhibit ABL kinase activity in human endothelial cells (EC). Human umbilical vein ECs were treated with the indicated BCR-ABL TKI or DMSO control at the Cmax concentration for 2 hours. **A.** Representative images of western blots for the ABL kinase target p-Y207 CRKL (top), total CRKL (middle) and β-actin loading control (bottom). **B.** Quantification of the ratio of p-CRKL to total CRKL. N = 4 independent experiments. One way ANOVA with Tukey’s multiple comparison test. *p<0.05, **p<0.01, ***p<0.001.

We first tested whether BCR-ABL TKIs inhibit native ABL kinase activity in human endothelial cells by quantifying the impact of the Cmax dose of each TKI on the Y207 phosphorylation site of CRKL, which is an ABL kinase specific phosphorylation target [35]. CRKL is a 39 kDa adaptor protein and important mediator of the oncogenic effects of BCR-ABL in leukemia cells [36] that is also expressed in endothelial cells [37]. Treatment of HUVECs with each TKI for 2 hours significantly decreased the level of p-Y207 CRKL normalized to total CRKL (Fig 1). For all figures, results from drugs with no known cardiovascular toxicity (imatinib) are indicated in blue, those with known cardiovascular toxicity (dasatinib, ponatinib, nilotinib) in red, and those with limited clinical follow up and hence unknown cardiovascular toxicity (asciminib, radotinib, bafetinib) are in purple. Despite different potencies of each drug, at the Cmax concentration used in patients, all of the drugs significantly inhibited ABL kinase compared to DMSO treated control cells with no statistically significant difference in the degree of inhibition of CRKL phosphorylation in HUVECs among any of the BCR-ABL TKIs tested.

### Vasculotoxic BCR-ABL TKIs impair human endothelial cell wound healing with differential impact on cell migration or proliferation

**Fig 2:**
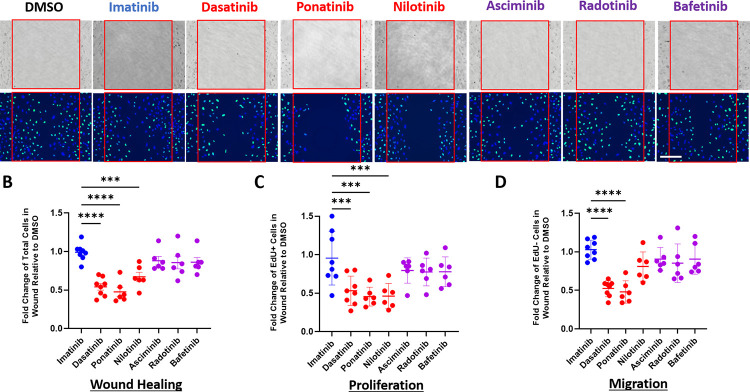
Vasculotoxic BCR-ABL TKIs, but not the novel TKIs, impair human endothelial cell wound healing. A scratch wound was made in a HUVEC monolayer and then cells were treated with the indicated BCR-ABL TKI or DMSO along with EdU. **A**. Representative light microscope images immediately after generating the scratch (top row) and fluorescent microscope images (bottom row) at 18 hours after the wound with staining for nuclei (blue) or Edu-incorporated nuclei (green). Red square highlights the wounded area. Scale Bar = 200 μM. **B.** Quantification of wound healing (total cells in the scratch relative to DMSO). **C.** Quantification of proliferation (EdU positive cells in the scratch relative to DMSO). **D**. Quantification of migration (EdU negative cells in the scratch relative to DMSO). **B-D.** Data are presented as mean ± SEM, with n = 6–8 independent experiments per treatment. One way ANOVA with Dunnett’s multiple comparisons test with comparison of each mean to imatinib. **p<0.01, ***p<0.001, ****p<0.0001.

To determine the impact of the BCR-ABL TKIs on endothelial cell wound healing, a scratch wound was created in a monolayer of HUVECs and the impact of each drug on cellular recovery in the wound was quantified 20 hours later and expressed as the fold change in cell count relative to DMSO control (Fig 2). Imatinib (the drug without increased risk of cardiovascular events) did not significantly impact wound healing, cell proliferation, or cell migration compared to DMSO (hence the value for Imatinib versus DMSO control is approximately 1). Treatment with dasatinib, ponatinib, or nilotinib (the three TKIs known to cause arterial thrombotic events) significantly decreased the relative amount of cells in the wounded area compared to imatinib (Fig 2B), consistent with prior studies [23]. Treatment with asciminib, bafetinib, and radotinib did not significantly impact wound healing compared to imatinib. HUVECs treated with dasatinib, ponatinib, and nilotinib also showed significantly decreased EdU positive cells in the wound compared to imatinib treated cells, supporting that these drugs impair EC proliferation (Fig 2C). Only dasatinib and ponatinib significantly decreased EdU negative cells in the wound (Fig 2D). As EdU negative cells in the scratch must have migrated from outside of the wound without proliferating, these data suggest that dasatinib and ponatinib also impair EC migration into the scratch wound. Asciminib, bafetinib, and radotinib did not significantly change the number of EdU-positive proliferated cells nor the EdU-negative migrated cells in the wound compared to imatinib (Fig 2C and 2D).

### Dasatinib, ponatinib, nilotinib, and bafetinib, but not asciminib or radotinib, decrease endothelial cell viability

**Fig 3:**
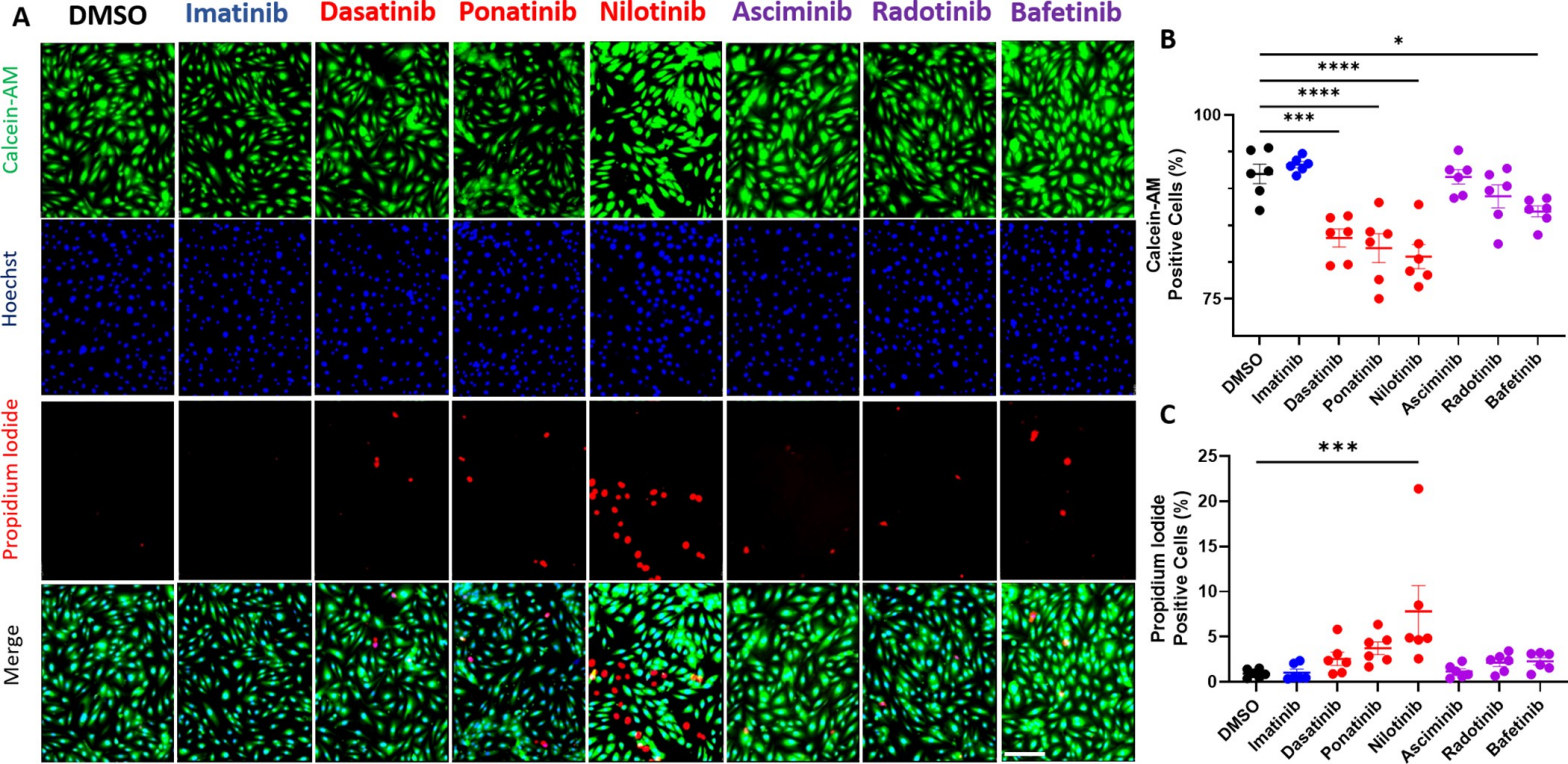
Dasatinib, ponatinib, nilotinib, and bafetinib, but not asciminib or radotinib, decrease endothelial cell viability. **A.** Representative images of HUVECs treated with each indicated TKI for 24 hours and stained for with calcenin-AM (viable cells), hoescht (all cells), and propidium iodide (necrotic cells). Scale Bar = 200 μM. **B.** Quantification of percent viable (calcein-AM positive/ hoescht positive) cells after treatment with each TKI or DMSO control as indicated. **C.** Quantification of percent necrotic (propidium iodide positive/ hoescht positive) cells after treatment with TKI or DMSO control as indicated. Data are presented as mean ± SEM, with N = 6 independent experiments per treatment. Data were analyzed with one-way ANOVA with Dunnett’s post-hoc test with comparisons of each condition to DMSO. **p<0.01, ***p<0.001, ****p<0.0001.

In addition to altering EC proliferation or migration, wound healing is impaired when EC survival is decreased. To test the impact on cell viability, HUVECs were treated with each BCR-ABL TKI for 24 hours and viable cells were marked by positive calcein-AM staining and necrotic cells marked with propidium-iodide and quantified relative to the total cell number determined by hoescht positive nuclei (Fig 3). Imatinib did not significantly impact cell viability or survival as there was no significant difference between imatinib treated and DMSO treated controls. Dasatinib, ponatinib, nilotinib, and bafetinib all significantly decreased the number of calcein-AM positive (viable) cells compared to vehicle (Fig 3B) providing the first evidence of EC toxicity from bafetinib. In further studies, dasatinib, ponatinib, and nilotinib also significantly decreased cell viability at 50% of the Cmax concentration, but not at 25% of the Cmax concentrations, while bafetinib only decreased viability at the Cmax (S1A Fig). Only nilotinib significantly increased the number of propidium-iodide positive necrotic cells (Fig 3C) and this toxicity was only observed at the Cmax dose, not at lower concentrations (S1B Fig). Asciminib and radotinib did not significantly change the percent calcein-AM or propidium iodide positive cells compared to DMSO (Fig 3B and 3C). The data thus far demonstrate that all of the BCR-ABL TKIs known to increase arterial thrombosis risk also impair EC wound healing, viability, and proliferation, and that dasatinib and ponatinib impair EC migration while nilotinib induces EC necrosis. Among the novel TKIs, asciminib and radotinib do not appear to cause EC dysfunction in these assays, while bafetinib causes a decrease in EC viability without increasing necrosis or impairing wound healing.

### Dasatinib and ponatinib impact both human endothelial cells and leukocytes to enhance leukocyte-endothelial adhesion

**Fig 4:**
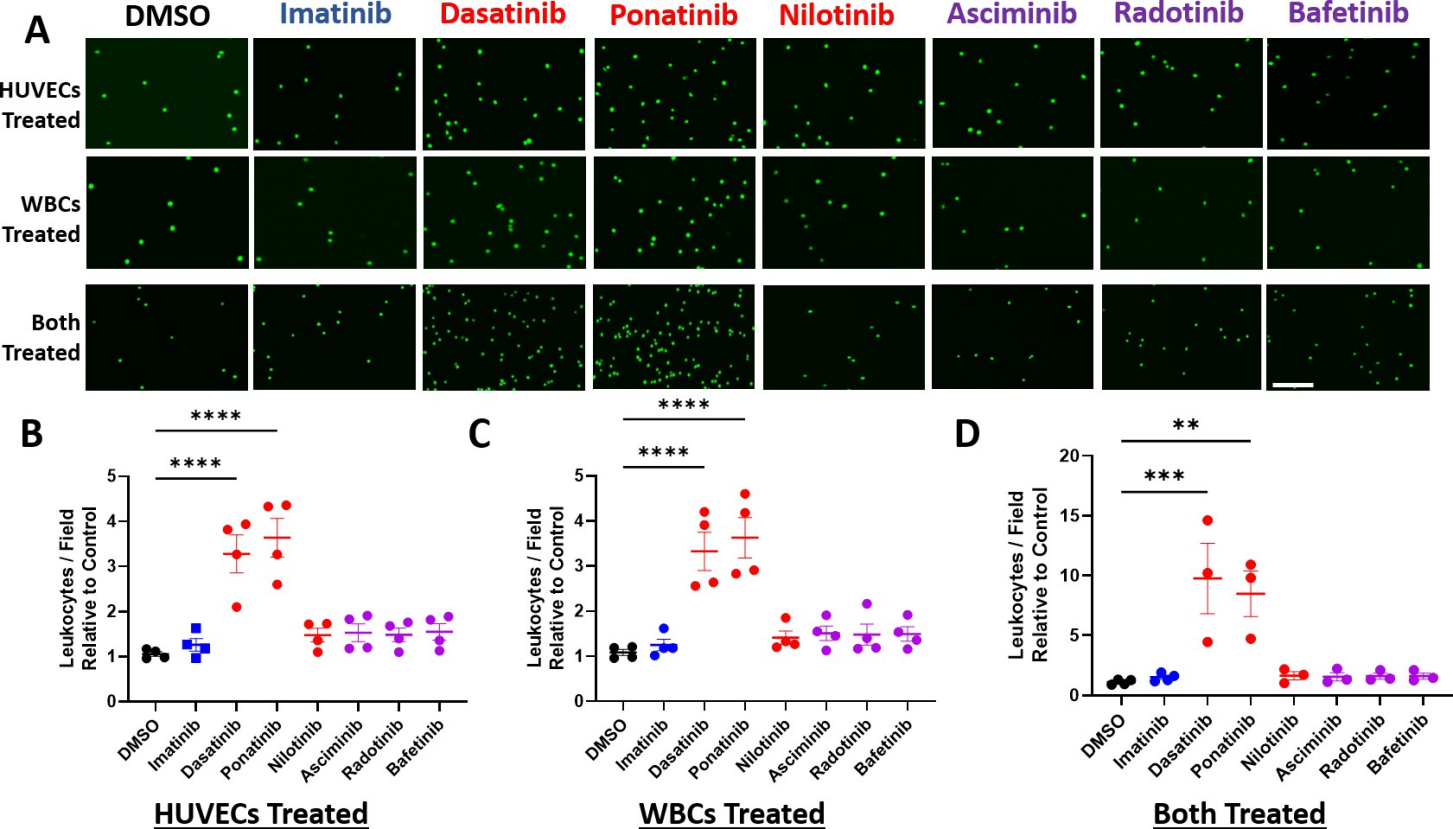
Dasatinib and ponatinib act on HUVECs and leukocytes to enhance leukocyte-endothelial cell adhesion. **A.** Representative images of U937 cells adherent to HUVECs after pre-treatment of HUVECs alone (top row), U937 cells alone (middle row), or pre-treatment of both cell types (bottom row). Scale Bar = 200 μM. **B.** Quantification of the number of adherent U937 cells relative to DMSO control after HUVEC pre-treatment with TKI. **C.** Quantification of adherent U937 cell relative to DMSO control after U937 leukocytes were pre-treated with TKI. **D.** Quantification of adherent U937 cells relative to DMSO control after both U937 cells and HUVECs were pre-treated with each TKI. Data are presented as mean ± SEM, with N = 3–4 independent experiments per treatment. Statistical analysis by one way ANOVA with Dunnett’s multiple comparisons test with comparison of each mean to DMSO. **p<0.01, ***p<0.001, ****p<0.0001.

Since toxic BCR-ABLTKIs are associated with acute MI and stroke, and rupture of inflamed atherosclerotic plaques is the predominant cause of MI and stroke, we next explored the impact of the BCR-ABLTKIs on vascular inflammatory mechanisms. The vasculature becomes inflamed when circulating leukocytes adhere to the damaged endothelium and migrate into the vessel wall. This leads to plaques with more inflammatory cells which are vulnerable to rupture, leading to arterial thrombosis, MI and stroke. Thus, we examined the impact of BCR-ABL TKIs on leukocyte adhesion to HUVECs, comparing the impact of treating only the HUVECs, only the leukocytes, or both cell types. There was no significant difference in leukocyte adhesion to HUVECs with DMSO treatment compared to imatinib treatment regardless of whether ECs, leukocytes, or both were treated (Fig 4). When HUVECs alone were treated with BCR-ABL TKIs for 24 hours and then incubated with fluorescently labeled U937 cells, dasatinib and ponatinib induced a significant 3- to 4-fold increase in adherent U937 cell adhesion compared to DMSO (Fig 4A). When only the U937 leukocytes were treated with TKIs and then incubated with untreated HUVECs, there was a similar 3- to 4-fold increase in the number of adherent cells with dasatinib and ponatinib versus DMSO (Fig 4C). Next, when U937 cells and HUVECs were both treated with TKIs prior to co-incubation, there was a synergistic increase in the amount of adherent U937 cells with dasatinib and ponatinib inducing a 9.8 fold and 8.5 fold increase in adherent leukocytes respectively. This suggested that these two BCR-ABL TKIs have direct pro-inflammatory effects on both leukocytes and endothelial cells. However, no increase in leukocyte adhesion was seen after treatment of either HUVECs, U937 cells, or both cell types with nilotinib, asciminib, bafetinib, or radotinib.

### Dasatinib and ponatinib induce expression of adhesion receptors on endothelial cells and adhesion ligands on leukocytes

**Fig 5:**
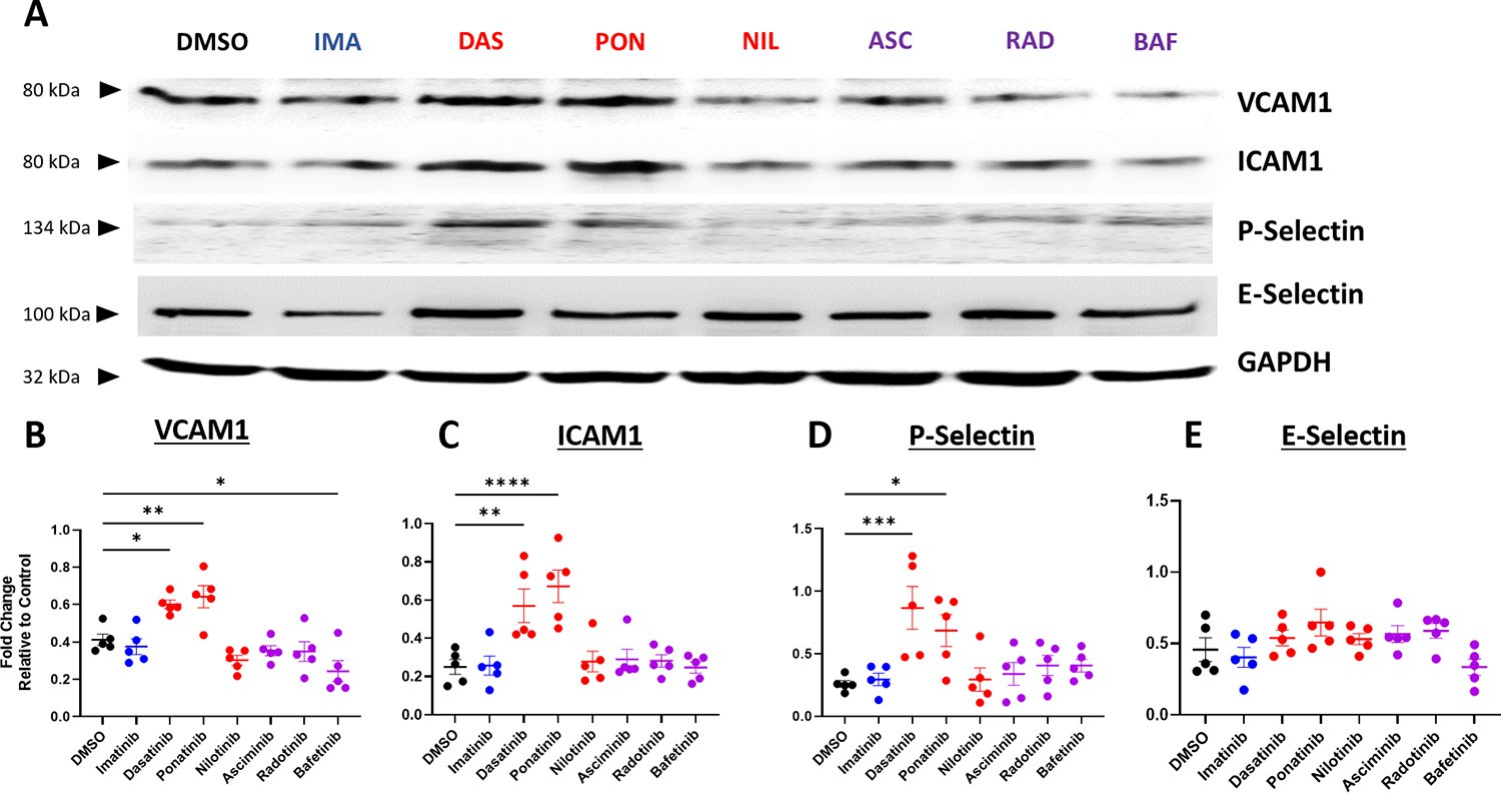
Dasatinib and ponatinib induce expression of leukocyte adhesion molecules on HUVECs. **A.** Representative western blots for VCAM1, ICAM1, P-Selectin, and E-Selectin of HUVECs treated for 24 hours with the indicated TKIs. **B-E.** Quantification of VCAM1 (B), ICAM1 (C), P-Selectin (D), and E-Selectin (E) relative to GAPDH after treatment with TKI or DMSO control. Data are presented as mean ± SEM, with N = 5 independent experiments per treatment. Statistical analysis by one way ANOVA with Dunnett’s multiple comparisons test with comparison of each mean to DMSO. *p<0.05, **p<0.01, ***p<0.001.

**Fig 6:**
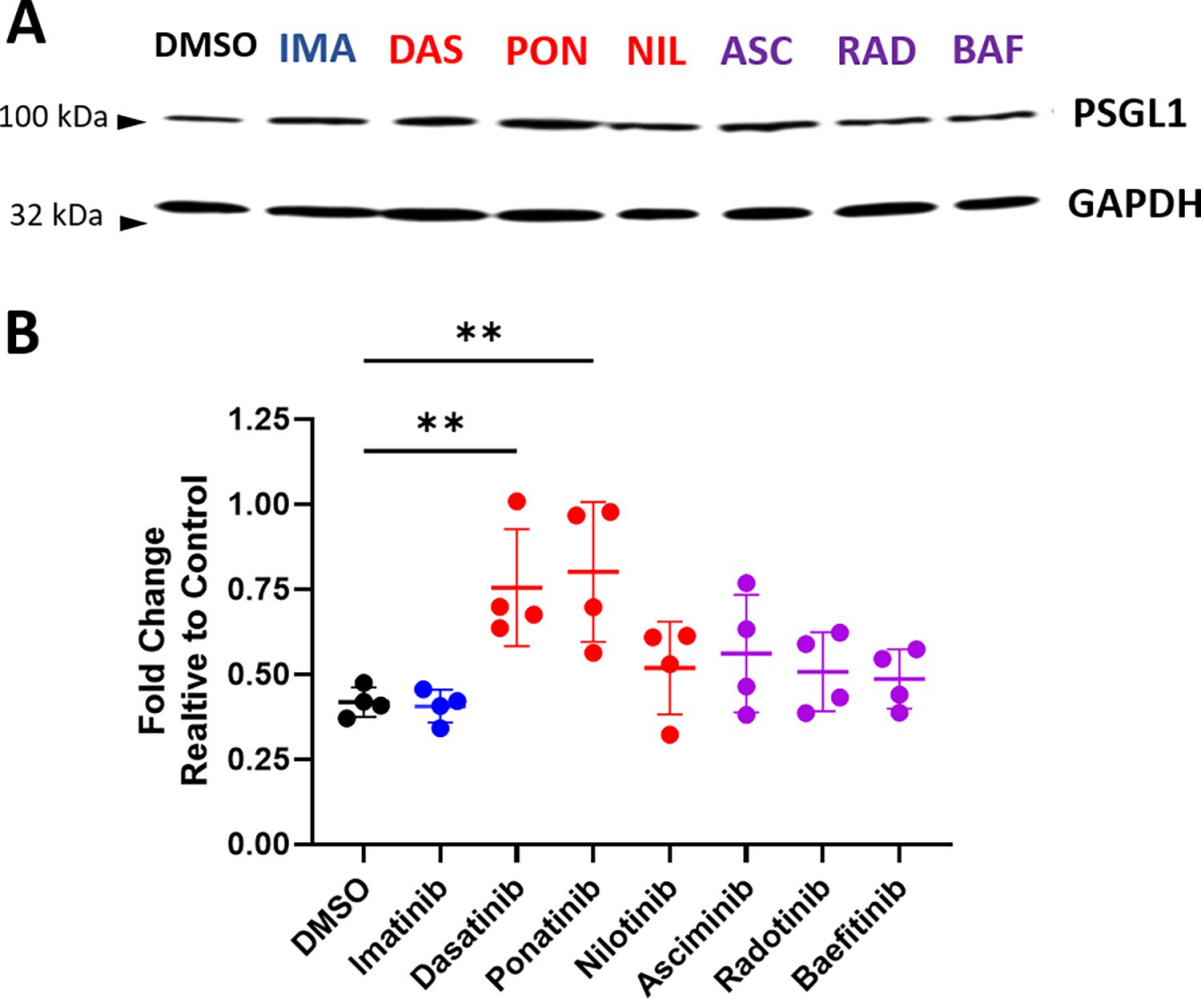
Dasatinib and ponatinib induce expression of the P-selectin glycoprotein ligand-1 on leukocytes. **A.** Representative western blot of U937 cells treated for 24 hours with the indicated TKIs. **B**. Quantification of PSGL1 relative to GAPDH loading control for cells treated with indicated TKI versus DMSO control. Data presented as mean ± SEM, with N = 4 independent experiments per treatment. Statistical analysis by one way ANOVA with Dunnett’s multiple comparisons test with comparison of each mean to DMSO. ** p < 0.01.

To explore the mechanism by which dasatinib and ponatinib induce leukocyte-endothelial cell adhesion, we examined the effects of BCR-ABL TKI treatment on adhesion molecule expression in HUVECs. Imatinib treatment did not significantly alter expression of any of the tested adhesion receptors on HUVECs or ligands on leukocytes compared to DMSO treated cells (Figs 5 and 6). Consistent with the leukocyte adhesion data, only treatment with dasatinib or ponatinib significantly increased the expression of ICAM1, VCAM1, and P-selectin whereas nilotinib, asciminib, bafetinib, and radotinib did not significantly alter leukocyte adhesion molecule expression(Fig 5A–5D). Bafetinib is the only TKI that significantly decreased VCAM1 expression (Fig 5B) without significantly affecting ICAM1 or P-selectin expression. E-selectin expression was not modulated by treatment with any BCR-ABL TKI tested (Fig 5A and 5E). Similarly, dasatinib and ponatinib treatment of the U937 cells significantly increased protein expression of the selectin ligand PSGL-1 compared to cells treated with DMSO, while there was no significant difference in U937 cells treated with imatinib, nilotinib, asciminib, bafetinib, or radotinib (Fig 6A and 6B).

### Dasatinib and bafetinib significantly increased permeability of human engineered microvessels (hEMVs) compared to imatinib

**Fig 7:**
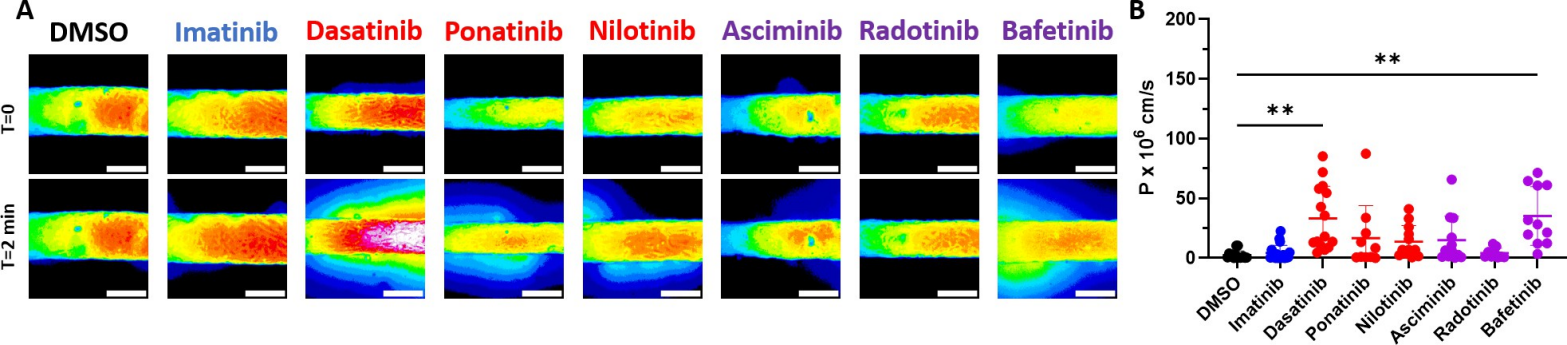
Dasatinib and bafetinib increase the permeability of human engineered microvessels. **A.** Representative images from T = 0 (top row) and T = 2 minutes (bottom row) of hEMVs treated with the indicated BCR-ABL TKI or vehicle control. **B.** Quantification of vessel permeability of hEMVs treated with each TKI or DMSO control. Data presented as mean ± SEM, with n = 9–19 independent experiments per treatment. Statistical analysis by Brown-Forsythe ANOVA with Dunnett’s T3 multiple comparisons test with comparison of each mean to DMSO. ** p < 0.01. Scale bar = 150 μM.

ECs form a tube lining the vessel with junctions between cells that prevent vascular leakage and leukocyte extravasation. Once leukocytes adhere to the damaged endothelium, they can transmigrate into the sub-endothelial space, a process that is facilitated by dissociation of cell-cell junctions that increases vessel permeability. Thus, we next examined EC permeability using a microfluidic platform in which a channel is formed within a collagen matrix and lined with HUVECs to create a perfusable 3-diminesional human engineered microvessel (hEMV). BCR-ABL TKIs were perfused through hEMVs for 3 hours followed by perfusion of 70 kDa Texas Red conjugated dextran. Transmural migration of the fluorescent dextran was then imaged and quantified (Fig 7). Imatinib did not significantly impact hEMV permeability compared to DMSO. Dasatinib (mean permeability coefficient 33.2 ± 6.9 cm/s) and bafetinib (mean permeability coefficient 35.2 ± 7.4 x 10^8^ cm/s) both significantly increased hEMV permeability compared to DMSO(mean permeability coefficient 2.47 ± 0.89 x 10^8^ cm/s), while treatment with ponatinib, nilotinib, asciminib, or radotinib did not significantly change hEMV permeability relative to DMSO (Fig 7).

### Dasatinib induces cytoskeletal and cell-cell junction alterations in human engineered microvessels (hEMVs)

**Fig 8:**
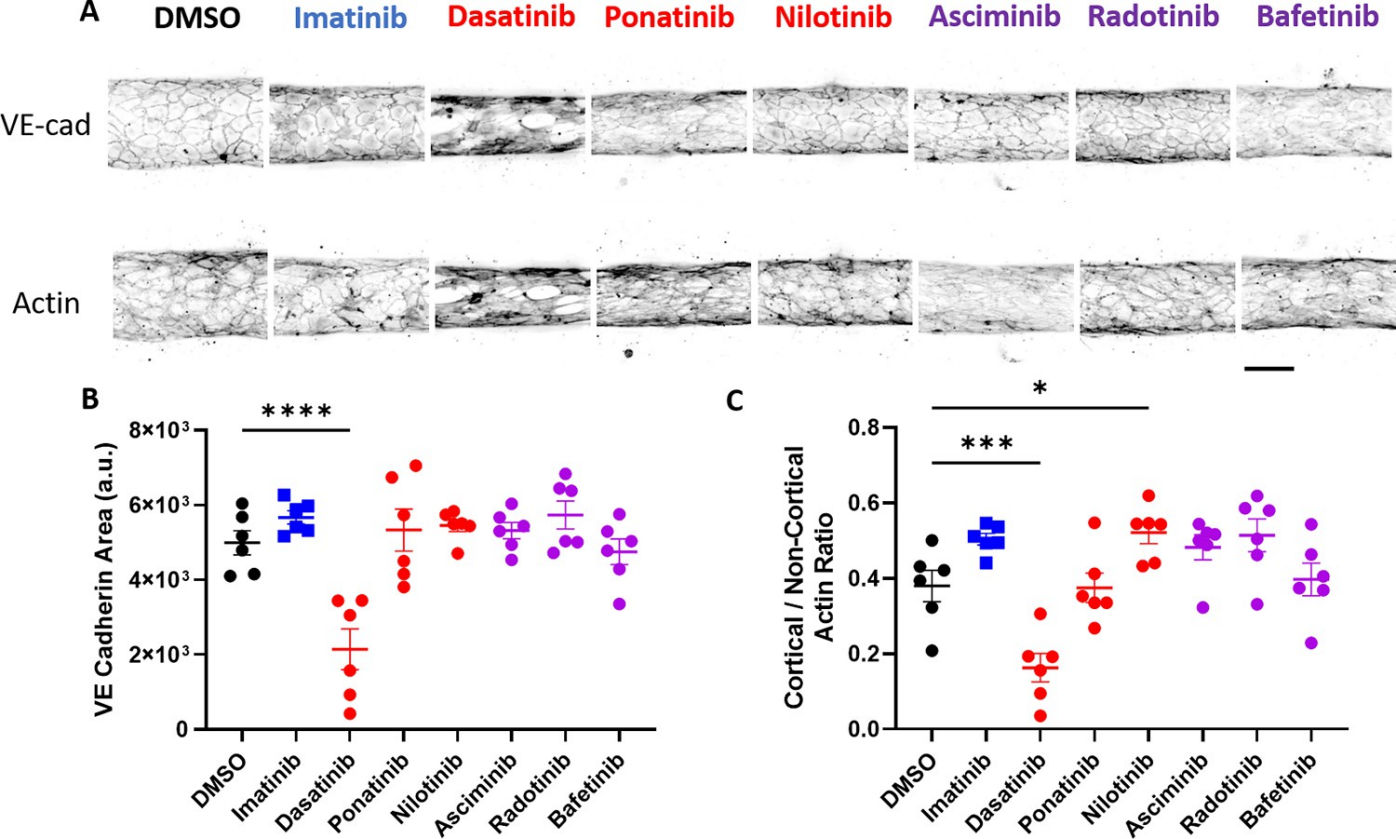
Dasatinib induces changes to the actin cytoskeleton and cell-cell junctions in human engineered microvessels (hEMVs). **A.** Representative images of hEMVs stained for VE-Cadherin (top row) or Actin (bottom row) after treatment with indicated BCR-ABL TKI or vehicle control for 3 hours. Scale bar = 150 μM. **B.** Quantification of VE-Cadherin staining for hEMVs treated with each TKI or DMSO control. **C.** Quantification of the ratio of junctional to cytoplasmic actin. Statistical analysis by one way ANOVA with Dunnett’s multiple comparisons test with comparison of each mean to DMSO. *, p <0.05, *** p < 0.001 **** p < 0.0001.

Endothelial cell adherens junctions are composed of cadherin proteins with links to the actin cytoskeleton that play a critical role in maintaining vessel barrier integrity [38]. Thus, to further investigate the mechanism by which BCR-ABL TKIs increase permeability of hEMVs, the treated hEMVs were fixed, and stained for VE-cadherin and actin (Fig 8A). There was no significant change in VE-cadherin positive area or junctional actin density for imatinib versus DMSO treated hEMVs. However, consistent with its marked increase in hEMV permeability, and a unique side effect in humans of pleural effusions, dasatinib significantly decreased VE-cadherin positive area and decreased the junctional actin intensity, suggesting that it increases vessel permeability through disruption of endothelial cell junctions and rearrangements in cytoskeletal structure (Fig 8B and 8C). Nilotinib significantly increased cortical/non-cortical actin ratio while not altering VE cadherin ratio, which is of uncertain importance given that it did not affect hEMV permeability (Fig 7). None of the other BCR-ABL TKIs caused significant changes to VE-cadherin area or cortical to non-cortical actin ratio, including bafetinib, despite the fact that it increased hEMV permeability.

## Discussion

This study directly compared for the first time the impact of seven BCR-ABL TKIs and vehicle on human EC functions *in vitro*, all at clinically relevant concentrations. This includes the 3 agents (dasatinib, ponatinib, and nilotinib) that are known to significantly increase the risk of acute arterial thrombosis and three recently developed drugs never before tested on ECs *in vitro* (bafetinib, asciminib and radotinib), and compared each to the first generation drug imatinib which does not induce arterial thrombosis risk. The data reveal that: 1) imatinib at the Cmax seen in patients does not significantly impact any of the EC function assays compared to DMSO control; 2) dasatinib, ponatinib, and nilotinib impair EC wound healing in the scratch wound assay with dasatinib and ponatinib decreasing both EC proliferation and migration, while nilotinib decreases EC proliferation only; 3) ponatinib, dasatinib, nilotinib, and bafetinib all decrease EC viability in a dose-dependent manner with nilotinib specifically inducing EC necrosis; 4) ponatinib and dasatinib increase leukocyte adhesion to the endothelial cells, with significant induction of adhesion molecule expression on both ECs and leukocytes while bafetinib decreases VCAM1 expression on HUVECS, and; 5) dasatinib, which is clinically associated with pleural effusion [39], and bafetinib (in phase I clinical trials) both significantly increase vascular permeability in hEMVs, potentially through rearrangements of adherens junctions where VE-cadherin links to the actin cytoskeleton.

These data are clinically significant for several reasons. First, the three drugs with known cardiovascular side effects all caused EC dysfunction *in vitro*, while imatinib did not. These data are consistent with previous studies from our lab in which we also showed that bosutinib, another second generation BCR-ABL TKI, did not impact HUVEC survival, wound healing, or leukocyte adhesion *in vitro* [23], and bosutinib has since been shown to lack cardiovascular side effects in patients [6]. This suggests that *in vitro* EC function assays may be able to predict vascular toxicity of drugs in humans and hence, the observation of a lack of EC toxicity of radotinib and asciminib in these assays may predict their safety from cardiovascular side effects. Conversely, the data here suggest close monitoring regarding bafetinib’s cardiovascular side effects as it progresses in clinical trials for CML and other malignancies, as it both decreased HUVEC viability and increased hEMV permeability in our studies. The importance of the fact that bafetinib significantly decreased VCAM1 expression in our studies is uncertain given its toxicity in other assays and lack of impact on leukocyte adhesion. Finally, we found that while all the drugs with side effects in humans cause EC dysfunction *in vitro*, the EC damage is mediated via overlapping yet not identical mechanisms. Thus, as we attempt to identify vasculoprotective strategies, our data suggest that a precision approach may be needed with distinct agents to address the side effects of different BCR-ABL TKIs.

Clinically, the high risk of arterial thrombosis with second and third generation BCR-ABL TKIs, and the development of pleural effusions caused by dasatinib, limits overall survival. Indeed, a meta-analysis showed that the benefits of second and third generation BCR-ABL TKIs in improving CML molecular remission are mitigated by the excess cardiovascular morbidity [6]. Thus, understanding the mechanism behind the cardiovascular toxicity of these drugs is an area of growing interest. The studies presented here add to the growing literature and understanding of EC toxicity as a major mediator of BCR-ABL TKI cardiovascular toxicity. Previous studies have investigated the EC toxicity of some of these BCR-ABL TKIs in several of the assays employed here but at concentrations that were not always clinically relevant and resulting in varied and often conflicting results (reviewed in [40]). In one study using the EA.hy926 hybridoma EC line, imatinib treatment decreased proliferation, increased apoptosis, and increased permeability, however the lowest concentration used in that study was 10 μm, which is 2.5 times higher than the C_max_ found in human plasma [41]. As reviewed by Hauget et al [40], multiple additional studies have shown that imatinib generally has no impact, or in some cases a protective effect, on EC function and survival, while dasatinib, ponatinib, and nilotinib all cause EC toxicity, as confirmed here. One previous study showed that nilotinib increased ICAM-1, VCAM, and E-selectin mRNA and protein levels in HUVECs and EA.hy926 cells [42] at very low doses (10 nM). The increase in adhesion molecules was attributed to IL-1β signaling, which correlated with increased IL-1β level in patients treated with nilotinib versus imatinib or dasatinib. This contrasts with the current findings that only dasatinib and ponatinib increased ICAM-1 and VCAM, while no TKI increased EC E-selectin protein level. It is possible that while low levels of nilotinib increase IL-1β signaling, at clinically relevant concentrations nilotinib causes other alterations that mitigate the increase in adhesion molecules seen at low concentrations or that other differences in experimental design underlie these discrepancies. Taken together, published data highlight the complexity of investigating the toxicities of kinase inhibitors as a therapeutic class and the importance of directly comparing the effects of multiple BCR-ABL TKIs, under similar conditions, and at clinically relevant concentrations.

An overarching question remains as to why different drugs that all inhibit ABL kinase have such varied cardiovascular toxicity, ranging from no evidence of toxicity (imatinib) up to temporary removal from the market and a black box warning from the FDA (ponatinib) due to an extremely high rate of arterial occlusion. The BCR-ABL TKIs inhibit ABL kinase with variable potency, leading to the use of very different doses to achieve cancer benefits, depending on the agent. Indeed the vasculotoxic TKIs are more potent ABL kinase inhibitors and dose reductions have been shown to reduced side effects of these agents [43,44] and endothelial toxicity (S1 Fig). It is possible that the vascular side effects are due to “on target” ABL inhibition in vascular cells that is enhanced with more potent agents. However, here we show that at the Cmax level seen in patients, all the BCR-ABL TKIs similarly inhibit EC ABL kinase (as measured by a decrease in phosphorylation of CRKL) including imatinib, asciminib, and radotinib, which do not induce EC toxicity *in vitro*. An alternative explanation comes from the knowledge that since all of the BCR-ABL TKIs target the kinase ATP-binding site (will the exception of the allosteric inhibitor asciminib), they also have a broad and varied range of “off-target” kinases that are also inhibited [40] that may also contribute to vascular toxicity. Indeed, a proteomic study demonstrated that the 3 vasculotoxic BCR-ABL TKIs induce a phospho-proteomic signature that is distinct from imatinib and predicted the safety of bosutinib [23]. Yet, examination of the kinase profiles for the various drugs does not implicate one specific kinase common only to dasatinib, ponatinib, and nilotinib that is not also shared with imatinib (and bosutinib) [19]. The studies presented here show that while all the vasculotoxic BCR-ABL TKIs impair EC function, they each have a distinct EC functional toxicity profile, providing further evidence for the concept that rather than one specific “off-target” kinase, it is likely a combination of different cellular perturbations secondary to multi-kinase inhibition that leads to important differences in the EC dysfunction phenotypes. For example, in this study dasatinib and ponatinib both impair EC viability, migration and enhance vascular inflammation, while nilotinib alone induced EC necrosis without pro-inflammatory effects and dasatinib alone also enhances permeability. Nilotinib is also unique in that it is associated with induction of traditional cardiac risk factors such as glucose intolerance and hyperlipidemia [45] and hence may also impact EC function *in vivo* secondary to effects on glucose or lipid levels that are not modeled in isolated ECs *in vitro*. The differential toxicity profiles have potential implications for clinical management. For example, nilotinib might be avoided in CML patients with poor metabolic control and the new data here suggests that TKIs such as dasatinib or ponatinib might be avoided in patients with known atherosclerosis or autoimmune diseases, as these conditions may be synergistic with the TKIs pro-inflammatory actions, a known driver of atherosclerotic plaque rupture. If endothelial inflammation and impaired wound healing are indeed major causes of increased risk of arterial thrombosis, then potential endothelial protective medications may be able to prevent arterial thrombosis in CML patients on TKIs better than the current mainstay of anti-platelet agents. This would be clinically important given that patients with hematologic malignancies are at high risk of bleeding as well as thrombosis [46]. Clinical trials would of course be needed to test these hypotheses.

This study also has potential clinical implications for CML patients with the T315I ABL kinase mutation (approximately 5% of CML [10]). In late 2021, the FDA granted asciminib breakthrough designation and expedited approval for patients with the T315I mutation. Prior to this, ponatinib was the only BRC-ABL TKI effective for this mutation [9,47]. As such, when ponatinib was pulled from the market in 2013 due to the high risk of arterial thrombotic events, it was rapidly reinstated with a black box warning because it was the only effective therapy for patients with T315I Philadelphia chromosome-positive leukemia. Asciminib is the first allosteric inhibitor of ABL kinase and hence functions by a distinct mechanism from all the other agents that bind to the ATP-binding pocket of ABL kinase [48]. With limited long term follow up, the cardiovascular safety of asciminib is not yet known. However, by directly comparing clinically relevant concentrations of asciminib to ponatinib, this study demonstrates that asciminib has no negative impact on human ECs in a wide range of assays in which ponatinib impairs EC function. While asciminib is currently only approved for those that already failed 2 other TKIs, this study might support testing of asciminib as a potentially safer first line agent for T315I Philadelphia chromosome positive leukemias.

This study has several limitations. First, while we compared many of the most clinically relevant TKIs head-to-head, for most assays we tested only one physiologically relevant concentration, the Cmax. We examined the impact of lower concentrations on HUVEC viability, showing that this toxicity is dose dependent and not observed at 25%- CMax for any BCR-ABL TKI tested. Further studies might include dose ranges based on these studies that could inform future clinical studies attempting to determine safe dose levels to reduce cardiovascular side effects. However as their clinical efficacy is also tied to dose, it is important to understand toxicities at all ranges as there are some patients who will require the FDA approved doses of these medications, or potentially even higher doses if tolerated. All drugs were dissolved in DMSO and compared to the same final concentration of DMSO of 0.1%. While we cannot rule out an impact of DMSO itself on HUVEC function, prior studies have suggested no substantial impact [49,50]. In addition, although the studies were performed *in vitro* using HUVECs, a primary human EC line that is commonly used to study vascular function, these cells likely do not completely model the ECs lining relevant vascular beds nor do they model all aspects of the cells of the typical CML patient, which is a 55–70 year old with underlying cardiovascular risk factors. Future studies could make use of other endothelial cell sources or primary cells from aged patients. Also, the studies here do not fully elucidate the molecular mechanisms responsible for the differential toxicity of the various BCR-ABL TKIs. Ongoing studies are planned to address this. Finally, while we performed these studies on human cells, including in engineered microvessels, further studies with animal models are needed to study the effects of BCR-ABL TKIs on endothelial cell functions *in vivo* to further investigate the potential link between endothelial cell toxicity, cardiovascular toxicity, and risk of thrombosis in the context of the whole organism.

## Conclusions

Despite these limitations, this study demonstrates that: 1) dasatinib, ponatinib, and nilotinib, and bafetinib all cause EC dysfunction *in vitro*; 2) asciminib, and radotinib do not cause EC toxicity *in vitro*, which corresponds to early clinical data showing no increase in adverse cardiovascular events with these agents [13]; 3) The toxic drugs damage endothelial cells via overlapping yet distinct mechanisms supporting precision medicine strategies for vasculoprotection. These findings increase our understanding of the diverse mechanisms of EC toxicity from BCR-ABL TKIs as mediators of their cardiovascular toxicity. Currently, our knowledge of whether a BCR-ABL TKI will induce cardiovascular toxicity is limited to waiting for long term follow up of clinical trials and post-marketing surveillance. The ability to predict cardiovascular toxicity from pre-clinical *in vitro* data would provide an advantage for drug developers screening for safer agents, clinicians trying to employ the safest treatments for patients, and could be used to help identify potential targets for protective intervention to limit the adverse vascular side effects of these medications.

## Supporting information

S1 Fig HUVEC toxicity of Dasatinib, Ponatinib, Nilotinib, and Bafetinib is dependent on dose. HUVECs were treated with the indicated BCR-ABL TKI or DMSO control at either Cmax, 0.5 Cmax (50% Cmax), or 0.25 Cmax (25% Cmax) concentration for 24 hours. A. Calcein-AM staining (viable cells) significantly decreased with dasatinib, ponatinib, and nilotinib cell viability at Cmax and 0.5 Cmax, but not at 0.25 Cmax while bafetinib only significantly decreases viability at Cmax. B. Propidium iodide staining (cell necrosis marker) shows that nilotinib significantly increased HUVEC necrosis at Cmax but not at lower concentrations. N = 4 independent experiments. One way ANOVA with Tukey’s multiple comparison test of all concentrations compared to each other. *p<0.05, **p<0.01, ***p<0.001, ****p<0.0001.
(TIF)

S1 Raw images (PDF)
