# Supplementary figures and images for: Differential vascular endothelial cell toxicity of established and novel BCR-ABL tyrosine kinase inhibitors
Source: PLoS One. 2023 Nov 20;18(11):e0294438. doi: 10.1371/journal.pone.0294438 (PMC10659179; doi:10.1371/journal.pone.0294438)

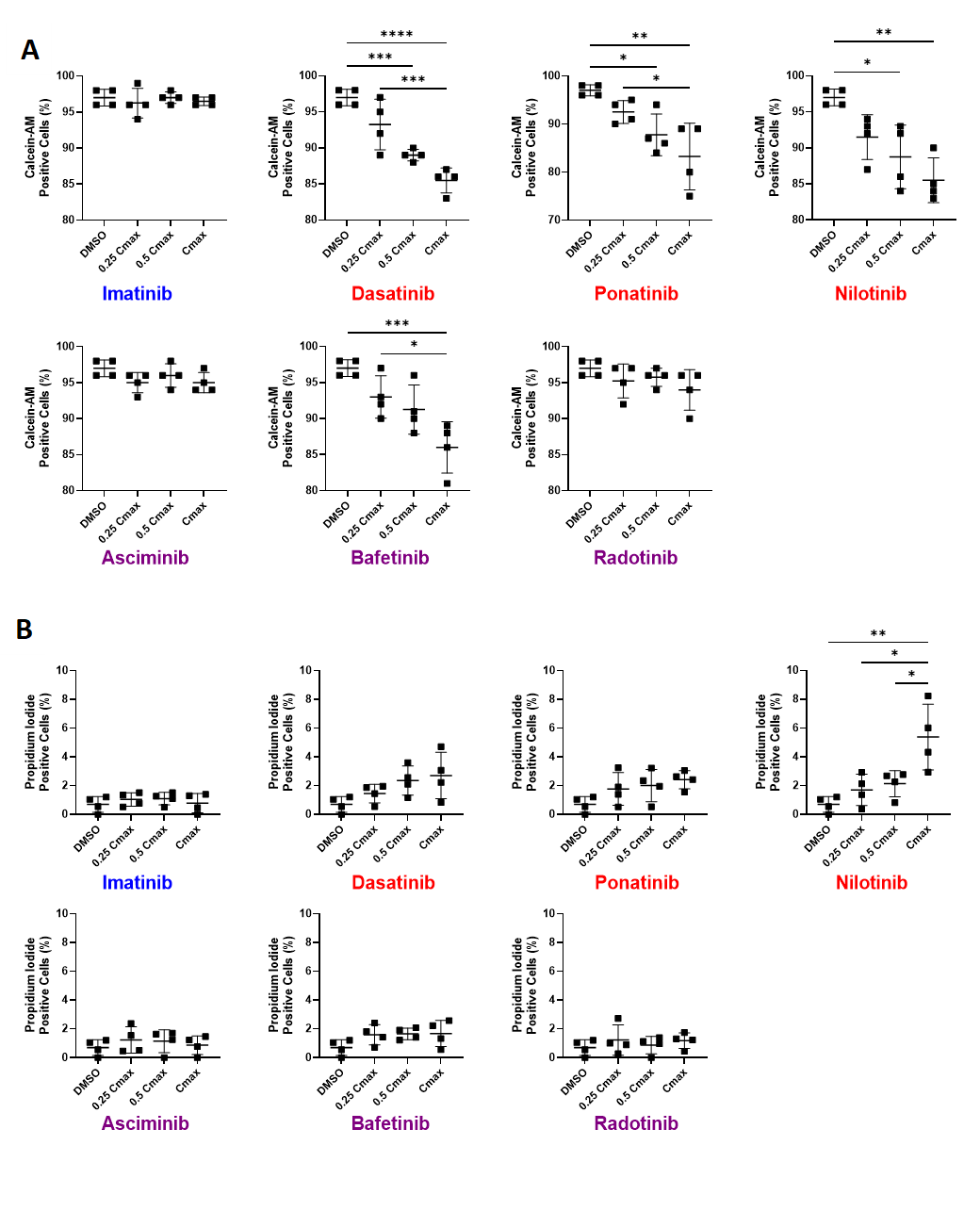

Supplement: S1 Fig — HUVECs were treated with the indicated BCR-ABL TKI or DMSO control at either Cmax, 0.5 Cmax (50% Cmax), or 0.25 Cmax (25% Cmax) concentration for 24 hours. A. Calcein-AM staining (viable cells) significantly decreased with dasatinib, ponatinib, and nilotinib cell viability at Cmax and 0.5 Cmax, but not at 0.25 Cmax while bafetinib only significantly decreases viability at Cmax. B. Propidium iodide staining (cell necrosis marker) shows that nilotinib significantly increased HUVEC necrosis at Cmax but not at lower concentrations. N = 4 independent experiments. One way ANOVA with Tukey’s multiple comparison test of all concentrations compared to each other. *p<0.05, **p<0.01, ***p<0.001, ****p<0.0001. (TIF) [file pone.0294438.s001.tif]
