# Supplementary material for: Differential vascular endothelial cell toxicity of established and novel BCR-ABL tyrosine kinase inhibitors
Source: PLoS One. 2023 Nov 20;18(11):e0294438. doi: 10.1371/journal.pone.0294438 (PMC10659179; doi:10.1371/journal.pone.0294438)
Supplement: S1 Raw images — (PDF) [file pone.0294438.s002.pdf]

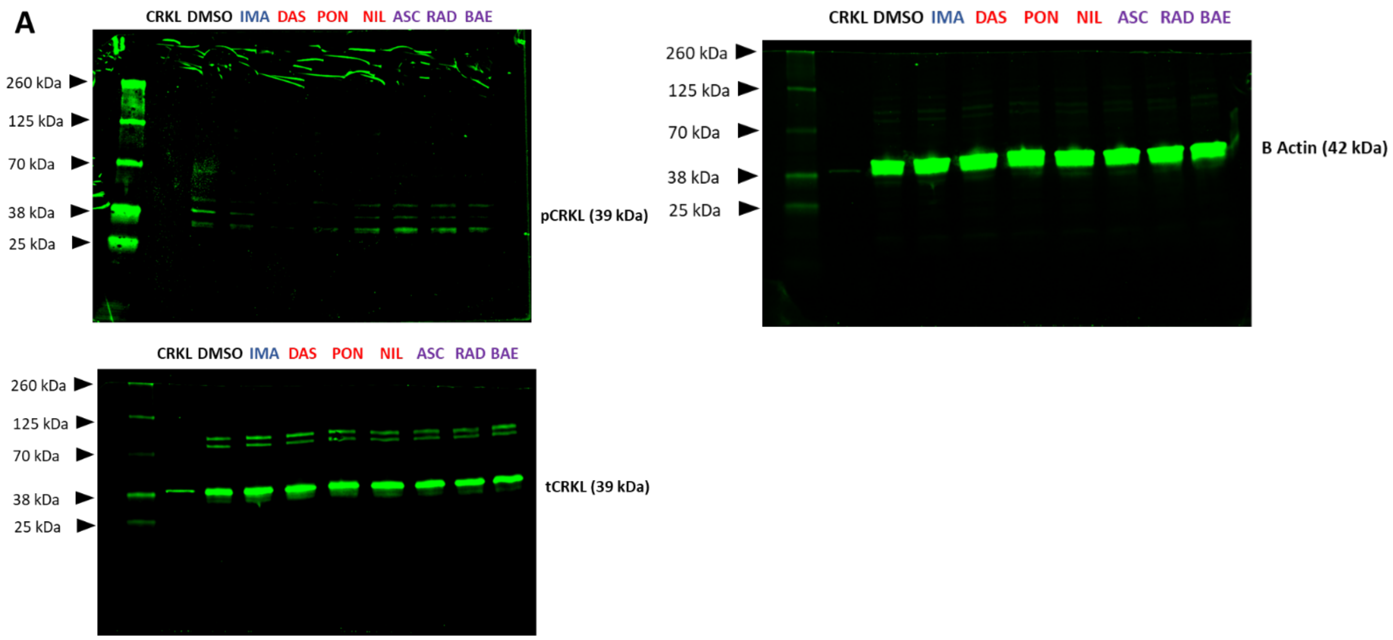

Uncropped western blot images from Fig 1 in manuscript. ABL kinase target p-Y207 CRKL (top left), total CRKL (bottom left) and B-actin loading control (top right). Imaged on a Licor Odyssey-M Imager and analyzed in Empiria Studio 2.3 (Licor).

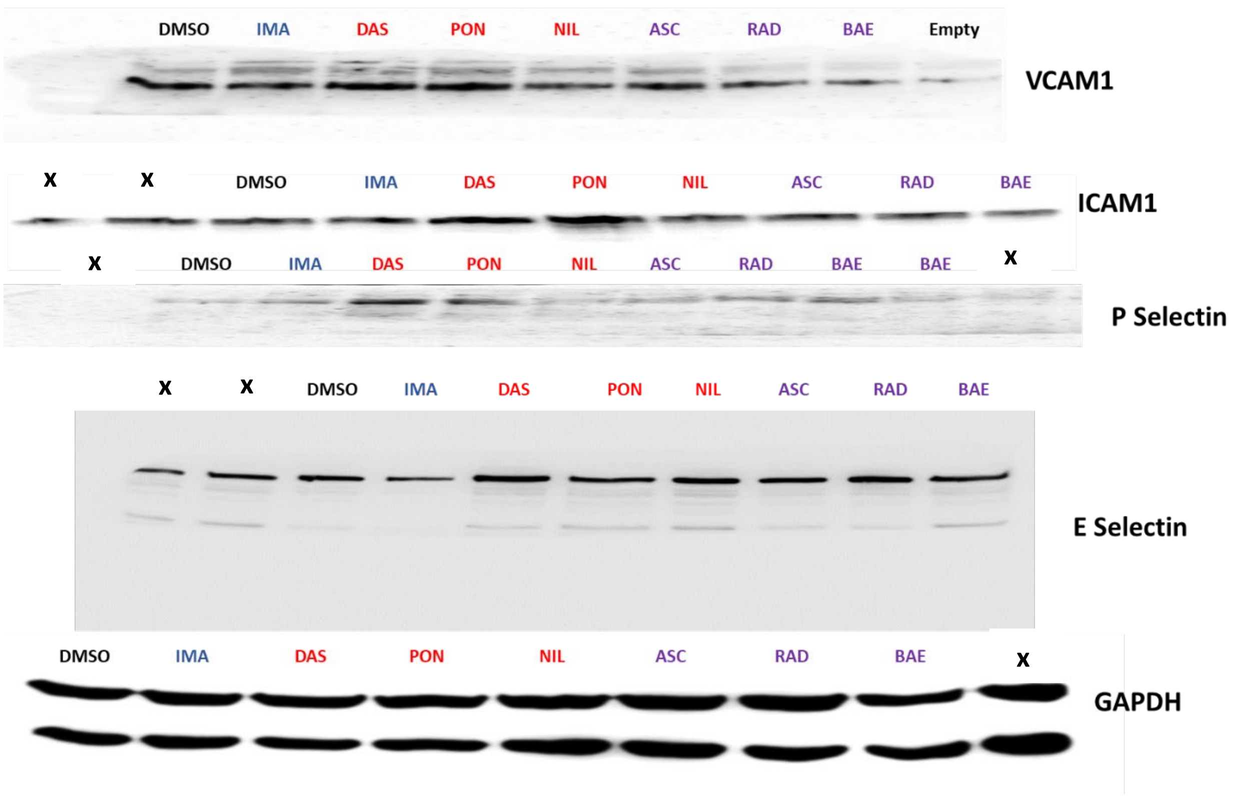

Uncropped western blots from Figure 5 in manuscript. Note that membranes were cut after transfer to enable staining of multiple proteins per blot. In places where duplicates were run, only one band per sample was quantified and included in quantification analysis. Imaged on FluorChem E imager (Bio-Techne) and analyzed with ImageJ.

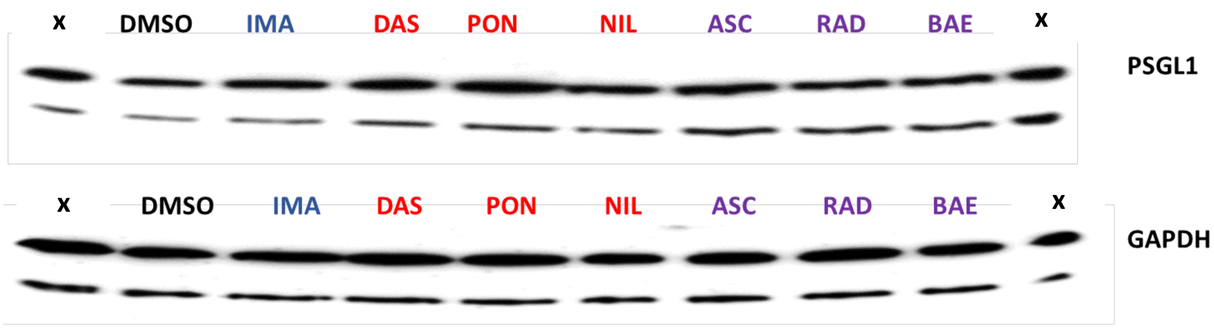

Uncropped western blot from Figure 6 in manuscript. Note that membranes were cut after transfer to enable staining of multiple proteins per blot. In places where duplicates were run, only one band per sample was quantified and included in quantification analysis. Imaged on FluorChem E imager (Bio-Techne) and analyzed with ImageJ.
